# The species richness pattern of vascular plants along a tropical elevational gradient and the test of elevational Rapoport's rule depend on different life‐forms and phytogeographic affinities

**DOI:** 10.1002/ece3.5027

**Published:** 2019-03-28

**Authors:** Yadong Zhou, Anne C. Ochola, Antony W. Njogu, Biyansa H. Boru, Geoffrey Mwachala, Guangwan Hu, Haiping Xin, Qingfeng Wang

**Affiliations:** ^1^ Wuhan Botanical Garden Chinese Academy of Sciences Wuhan China; ^2^ Sino‐Africa Joint Research Center, Chinese Academy of Sciences Wuhan China; ^3^ University of Chinese Academy of Sciences Beijing China; ^4^ East African Herbarium, National Museums of Kenya Nairobi Kenya

**Keywords:** Africa, elevation, plants, Rapoport's rule, tropical mountain

## Abstract

The research about species richness pattern and elevational Rapoport's rule (ERR) have been carried out mostly in the temperate regions in the recent years and scarcely in the tropical mountains; meanwhile, it is unclear whether the ERR is consistent among different life‐forms and phytogeographic affinities. Here, we compiled a database of plant species of Mount Kenya, a tropical mountain of East Africa, and divided these species into twelve groups depending on the life‐form and phytogeographic affinity of each species. We inspected the species richness pattern of each group along the elevation gradient and also tested ERR of each group using Stevens' method. Our results showed that species richness of the total species showed a positively skewed (hump‐shaped) pattern along the elevation gradient and different life‐forms and phytogeographic affinities showed similar hump‐shaped patterns as the total species. The average elevation range size of the total species and herbaceous species showed increasing patterns along the elevation gradient, while lycophytes and ferns, and woody species showed an obvious downward trend after peaking in the high elevation regions. We concluded that the widely distributed herbaceous species which also have broad elevation range sizes are more applicable to ERR, while the narrowly distributed woody species with small elevation range sizes occurring in the higher elevations could reverse ERR. Therefore, we concluded that the ERR is not consistent among different organisms in the same region.

## 1 INTRODUCTION

Understanding biodiversity patterns along the elevational gradients have been a hot topic of debate for decades between biogeographers, ecologists and biodiversity conservationists (Lomolino, 2001). Mountains are the ideal natural experimental environments for the study of species richness variety along the elevation gradients, because they not only harbor vast biodiversity and encompass several protected areas (Khan, Page, Ahmad, & Harper, 2014; Kluge et al., 2017; Körner, 2000, 2007; Smith, Oca, Reeder, & Wiens, 2007), but also because they contain diverse elevation gradients along their slopes (McCain, 2009; Rahbek, 2005; Stevens, 1992) which directly or indirectly impact the variations in availability of essential resources such as heat energy and moisture (Körner, 2000), affecting the physiological and ecological adaptation of plants thus influencing their species richness and patterns of distribution along the elevation gradients (Kessler, 2000; Kluge & Kessler, 2011; Lomolino, 2001).

Biodiversity patterns along the elevation gradients have been documented for numerous taxa and topographical extents (Rahbek, 1997; Rahbek & Museum, 1995; Stevens, 1992; Vetaas & Grytnes, 2002; Wu et al., 2014). Generally, positively skewed (hump‐shaped) and monotonically decreasing are the two most common patterns of species richness along the elevation gradients of mountains (Rahbek, 2005; Rahbek & Museum, 1995). The former pattern means species richness increases firstly, then decreases after the mid‐altitude peak, and the maximum diversity occurs below the middle of the elevation gradients (Kessler, 2000; Shmida & Wilson, 1985; Trigas, Panitsa, & Tsiftsis, 2013; Vetaas & Grytnes, 2002). The latter pattern means species richness decreases gradually along the elevation gradients (Kikkawa & Williams, 1971; Odland & Birks, 1999; Patterson, Pacheco, & Solari, 1996; Stevens, 1992; Tinner & Theurillat, 2003). Beyond that, few other patterns of species richness‐elevation gradients, such as increasing or horizontal, followed by a decreasing pattern were also reported (Brehm, Süssenbach, & Fiedler, 2003; Machac, Janda, Dunn, & Sanders, 2011; Rahbek, 2005; Rahbek & Museum, 1995).

Rapoport's rule, being the second robust biodiversity rule, is the positive relationship of species range sizes with the increasing biogeographic gradients, such as latitude, elevation, or water depth (Stevens, 1989, 1992, 1996). The latitudinal and elevational Rapoport's rules are the most examined in the literature, and there is a high degree of variability in support from supportive (e.g., latitudinal: (Arita, Rodríguez, & Vázquez‐Domínguez, 2005; Blackburn & Gaston, 1996; Luo et al., 2011, and elevational: Feng, Hu, Wang, & Wang, 2016; Patterson et al., 1996; Ribas & Schoereder, 2006; Rohner et al., 2015; Sanders, 2002;) to little or no support (e.g., latitudinal: Reed, 2003; Ribas & Schoereder, 2006; Rohde, Heap, & Heap, 1993; Rohde, 1996, and elevational: Bhattarai & Vetaas, 2006; Fu, Wu, Wang, Lei, & Chen, 2004; McCain & Knight, 2013; Rahbek, 1997).

The core prediction of elevational Rapoport's rule (ERR) is a positive and linear relationship between average elevation range size of species within increasing bands of elevation, which has subsequently been named as Stevens' method (Stevens, 1992); however, the range size‐elevation patterns of different taxonomic groups may be different (Feng et al., 2016; McCain & Knight, 2013). The life‐forms of plants are the response of plants to adapt to the eco‐physiological traits to climatic or environmental factors. Herbaceous and woody taxa are believed to be differentially influenced by environmental factors such as precipitation and temperature (Whittaker, 1965). The species richness of different life‐forms of plants always shows the similar hump‐shaped pattern along the elevation gradients with different peaks at intermediate elevations (Kluge et al., 2017). Nevertheless, that is not to imply that the range‐elevation relationships of different life‐forms will be consistent. In addition, phytogeographic affinities may be linked with elevational range sizes and their elevational trends (Feng et al., 2016; Wang, Tang, & Fang, 2007), that is, compared with narrowly distributed species, widely distributed ones always have broader tolerance ranges and stronger adaptability (Donohue, Rubio, Burghardt, Kovach, & Willis, 2010; Gaston & Spicer, 2001; Santamaría, 2002). However, in recent studies, little attention has been paid to compare the difference of ERR with regard to life‐forms and the influence of phytogeographic affinities.

Compared to the tropics, numerous studies about ERR have been carried out in the temperate regions in the recent years (Acharya, Vetaas, & Birks, 2011; Bhattarai & Vetaas, 2003; Kessler, Herzog, Fjeldså, & Bach, 2001; Kluge et al., 2017); furthermore, the support of the rule is scarce in the tropics (Gaston, Blackburn, & Spicer, 1998; Rohde, 1996). Evaluating and determining the patterns of species richness along the elevation gradients in the tropics is crucial as threats to the tropical biodiversity, currently, at risk of extinction, are snowballing due to destructive anthropogenic activities and the ongoing global warming predicament. Mount Kenya is the second highest mountain in tropical East Africa which has huge biodiversity and possesses a wide range of elevation gradients with fluctuating climatic conditions. Its gradients imitate the arrangement of species from the tropics to the poles at the local scale as the species occupy their particular elevational zones.

Our study is the first on Mount Kenya dealing with the statistical determination of plants elevation range sizes of different life‐forms and different phytogeographic affinities along the elevation gradient. This study aims to respond to the ensuing queries: (a) does species richness decrease with increasing elevation or there is a peak at an intermediate elevation? and (b) do the different range‐elevation relationships vary with the life‐forms and phytogeographic affinities?

## 2 MATERIALS AND METHODS

### 2.1 Study area

**Figure 1:**
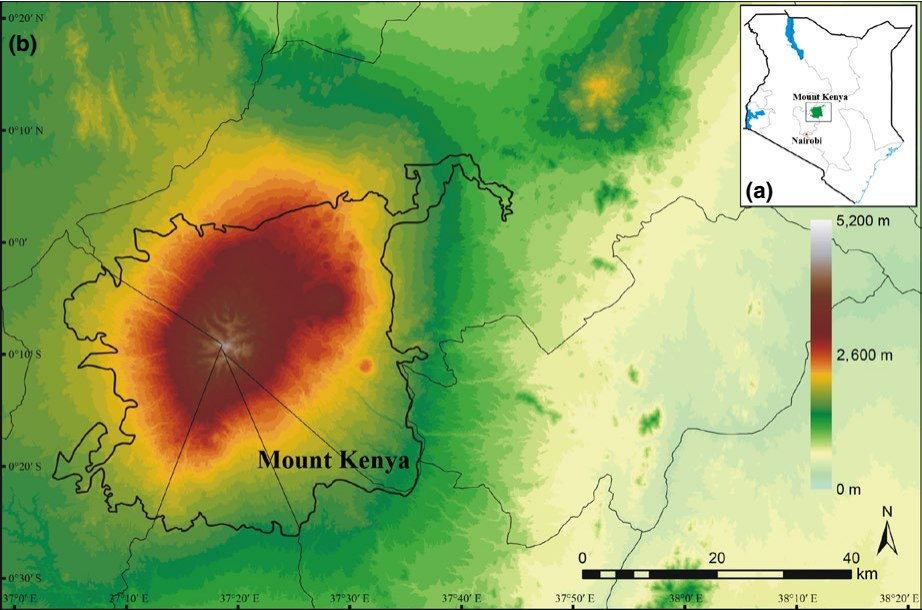
The map of Mount Kenya. (a) the location of Mount Kenya in Kenya; (b) the elevation map of Mount Kenya, showing 100‐m vertical elevation bands

Mount Kenya (0°10'S, 37°20'E) straddles the equator and is located in the central part of Kenya, about 193 km northeast of Nairobi and 480 km from the Kenyan coast (Figure 1a). The Lower Imenti Forest Reserves, located in the northeast past of Mount Kenya (Gathaara, 1999), are the lowest regions with an altitude of about 1,200 m a.s.l.; in addition, few plants can survive near the glacier above 5,000 m a. s. l. of this mountain (e.g., F.T.E.A. editors, 1952; Agnew, 2013). To examine the relationship between species richness and elevation range size of vascular plants along the elevation gradient of Mount Kenya, the total elevation ranges from 1,200 to 5,000 m a. s. l. was divided into 38, 100‐m vertical elevation bands (Figure 1b).

### 2.2 Plant data sources

A checklist of Mount Kenya containing 1,477 indigenous vascular plants including subspecies and varieties (belonging to 157 families and 686 genera) were compiled, based on the data collected during numerous scientific expeditions in this region since the 1900s: data from published monographs and field guides including *Flora of Tropical East Africa*, *Upland Kenya Wild Flowers and Ferns*, *Wild Flowers of East Africa*and *Kenya Trees Shrubs and Lianas* (Agnew, 2013; Beentje, 1994; Blundell, 1987; F.T.E.A. editors, 1952), data of specimens from the East African Herbarium, Nairobi, Kenya (EA) and Global Biodiversity Information Facility (GBIF, https://www.gbif.org/), and data from our own collections from 2009 to 2016 with specimens stored at the Herbarium of Wuhan Botanical Garden, Wuhan, China (HIB).

### 2.3 Life‐forms

Following Zhou et al. (2018), life‐form of each species was classified as woody plants (trees, shrubs, lianas) and herbaceous plants (climbers and herbs) and lycophytes and ferns based on the species description on monographs and field guides (Agnew, 2013; Beentje, 1994; Blundell, 1987; F.T.E.A. editors, 1952).

### 2.4 Phytogeographic affinities

According to the distribution range of each species, we set up three groups of phytogeographic affinities: worldwide species which are distributed not just in Africa, African species which are endemic in Africa, and tropical East African species which are endemic in Kenya, Uganda, Tanzania, and their vicinities. We also recorded the endemic species of Mount Kenya.

### 2.5 Species richness

The number of species present in each band was estimated by the interpolation method, that is, a species was defined as being present in every 100‐m elevation band between its upper and lower elevation limits (Bhattarai & Vetaas, 2006; Rahbek, 1997; Vetaas & Grytnes, 2002). The species richness was defined as the total number of species found in each 100‐m elevation band, referred to as γ‐diversity (Bhattarai & Vetaas, 2006; Lomolino, 2001). We calculated the species richness‐elevation patterns of the total plants, each life‐form and each group of phytogeographic affinities.

### 2.6 Elevation range size

The elevation range of each species was estimated as the difference between the maximum and minimum elevations, whose data were from literatures, specimens, and our own field observations. Actually, several methods have been frequently used in the recent decades to evaluate ERR, such as Stevens' method (Stevens, 1989), the midpoint method (Rohde, 1992), Pagel's method (Pagel, May, & Collie, 1991), and the cross‐species method (Letcher & Harvey, 1994), and often provide information that complements different perceptions of the patterns. In order to compare the results of different components under the same standard, we exclusively used Stevens' method to investigate the average range size‐elevation patterns of each group (including total, lycophytes and ferns, woody, herbaceous, trees, shrubs, lianas, climbers, herbs, worldwide, African and Tropical East African species) along the elevation gradient of Mount Kenya. We used generalized additive models (GAM) with a Gaussian function of variance to determine the trends of the response curve of species richness and range size along the elevation gradient, instead of using linear correlation analysis (Bhattarai & Vetaas, 2006; Feng et al., 2016). In this method, a cubic smooth spline was used to evaluate the significance of a specific trend for species richness‐elevation and range size‐elevation relationships (Hastie & Tibshirani, 1990). These analyses were carried out using R 3.3.3 software (R Core Team, 2017).

## 3 RESULTS

### 3.1 Species richness along the elevation gradient

**Figure 2:**
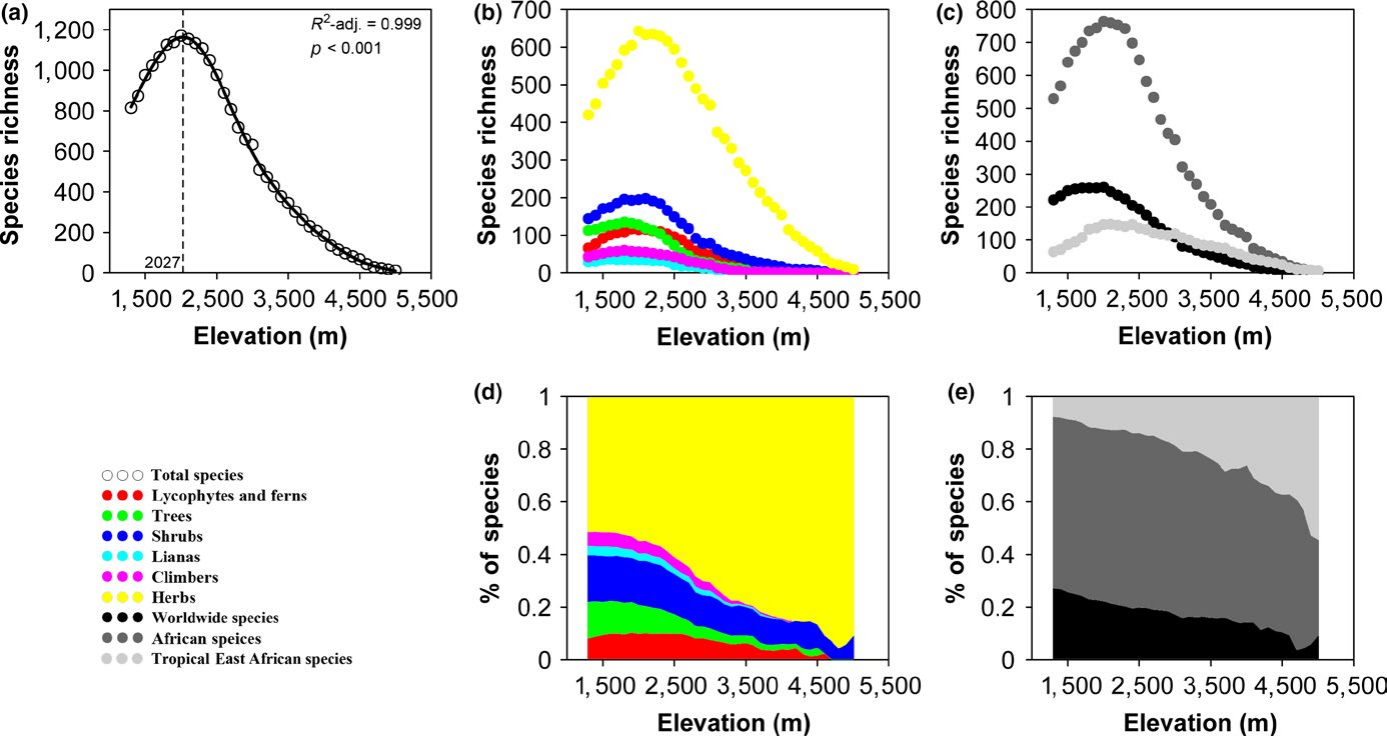
Elevational trends of species richness of vascular plants of Mount Kenya. (a) total species; (b) different life‐forms (lycophytes and ferns, woody, herbaceous, trees, shrubs, lianas, climbers, and herbs); (c) different phytogeographic affinities (worldwide, African, and tropic East African species); (d) the proportion of different life‐forms; and (e) the proportion of different phytogeographic affinities

Species richness of the total species showed a positively skewed (hump‐shaped) pattern along the elevation gradient, with a pronounced mid‐elevational peak at 2027 m a.s.l. containing over 1,000 taxa in each band of this range; meanwhile, there were species in less than 100 taxa above 4,300 m a.s.l. in each band, and only 11 species were found around 5,000 m a.s.l (Figure 2a). Different life‐forms showed similar hump‐shaped patterns as the total species (Figure 2b), with the proportion of woody species decreasing while the proportion of herbaceous species increased along the elevation gradient (Figure 2d). Meanwhile, different groups of phytogeographic affinities also showed similar hump‐shaped patterns as other groups (Figure 2c), with the proportion of worldwide species decreasing while the proportion of tropical East African species increased along the elevation gradient (Figure 2e).

### 3.2 Endemism along the elevation gradient

**Figure 3:**
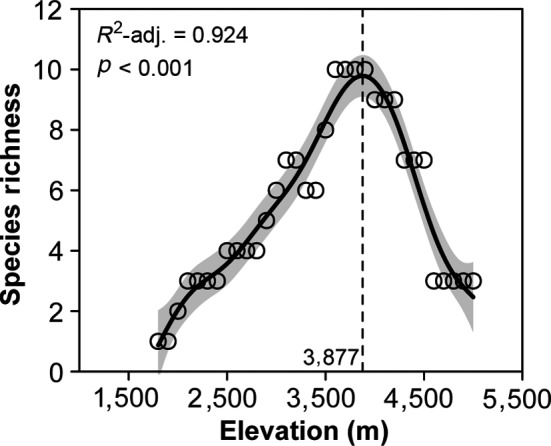
The species richness‐elevation pattern of endemic species of Mount Kenya

There were no endemic species of Mount Kenya below 1800 m; in contrast to the species richness‐elevation patterns of total species, endemic species were concentrated at the upper end of the elevational gradient with the highest values at about 3,900 m (Figure 3).

### 3.3 Elevation range size

**Figure 4:**
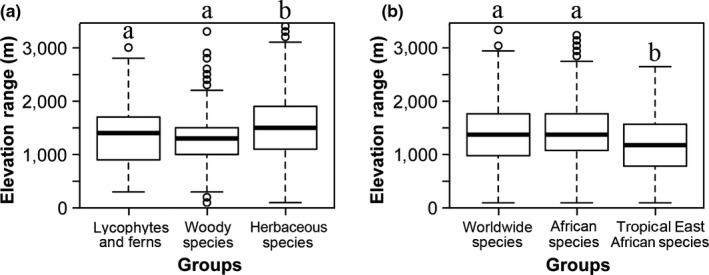
Comparison of elevation range between different groups regardless of the elevation gradient. (a) elevation range of lycophytes and ferns, woody, and herbaceous species; (b) elevation range of worldwide, African, and tropic East African species. The letters indicate significant differences (*α* = 0.05) between different groups

Regardless of the elevation gradient, we first compared the elevation range of life‐forms (including lycophytes and ferns, woody, and herbaceous species) and phytogeographic affinities (worldwide, African, and tropical East African species) (Figure 4). The elevation range of herbaceous species was significantly higher than that of lycophytes and ferns and woody species, while there was no significant difference between the latter two groups (Figure 4a). Meanwhile, the elevation range of the tropical East African species was significantly lower than the worldwide and African species, while there was no significant difference between the latter two groups (Figure 4b).

### 3.4 Mean elevation range size along the elevation gradient

**Figure 5:**
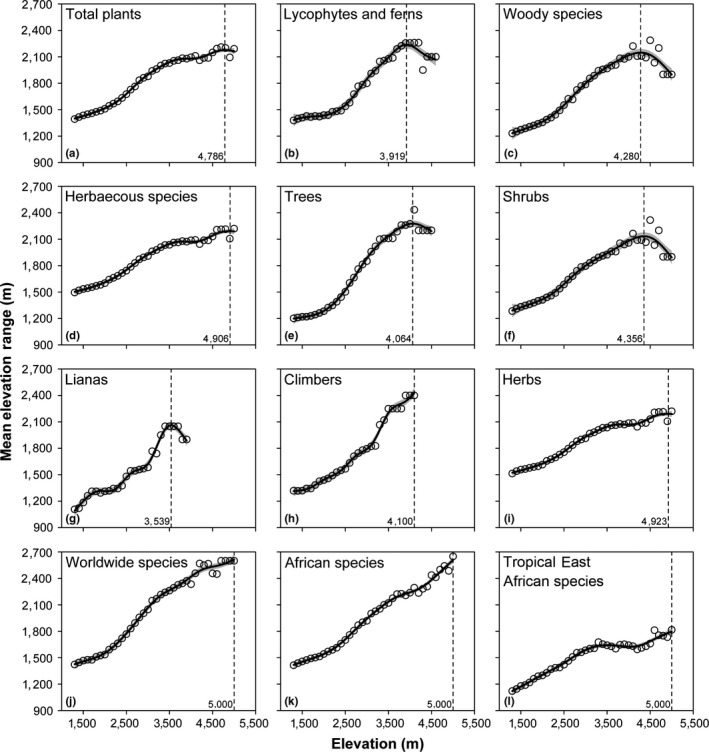
The mean elevation range of different group of species along the elevation gradient of Mount Kenya. (a) total; (b) lycophytes and ferns; (c) woody species; (d) herbaceous species; (e) trees; (f) shrubs; (g) lianas; (h) climbers; (i) herbs; (j) worldwide species; (k) African species; (l) tropical East African species. The effective degrees of freedom (edf), *R* ^2^‐adjusted and *p*‐values of each group showed in Table 1

Calculated herein are the mean elevation range size of 12 groups of species including total species, lycophytes and ferns, woody species, herbaceous species, trees, shrubs, lianas, climbers, herbs, worldwide species, African species, and tropical East African species. In general, the average elevation range size of all these 12 groups of species showed increasing patterns along the elevation gradient, while lycophytes and ferns, woody species, trees, shrubs, and lianas showed an obvious downward trend after peaking in the high elevation regions (Figure 5).

**Table 1:** Summary of generalized additive models used to describe the relationship between mean elevation range size and elevation of different groups

Groups	Effective degrees of freedom	*R^2^*‐adjusted	*p*‐values
Total species	7.864	0.993	<0.001
Lycophytes and ferns	7.109	0.978	<0.001
Woody species	5.886	0.974	<0.001
Herbaceous species	7.800	0.990	<0.001
Trees	6.039	0.991	<0.001
Shrubs	6.226	0.965	<0.001
Lianas	7.976	0.987	<0.001
Climbers	8.076	0.991	<0.001
Herbs	7.742	0.990	<0.001
Worldwide species	7.095	0.993	<0.001
African species	6.722	0.995	<0.001
Tropical East African species	6.397	0.979	<0.001

## 4 DISCUSSION

Mountains are usually more likely to display unimodal patterns for they invariably exhibit greater elevational extent and longer climatic gradients. In fact, most of the unimodal gradients were positively skewed (hump‐shaped), that is, peak diversity below the elevational midpoint, and this pattern is a well‐known finding for many tropical and subtropical mountains (Guo et al., 2013; Rahbek, 2005; Rahbek & Museum, 1995). Global data research showed that over 80% of species richness‐elevation patterns occurring in the tropical mountains are unimodal (Guo et al., 2013). The species richness of vascular plants of Mount Kenya also showed a strong support for the positively skewed pattern along the elevation gradients, with the maximum diversity at about 2000 m a.s.l., below the middle of the elevation gradients. These results emanate from the empirical data, which have been compiled by us based on collections from numerous scientific expeditions since the 1900s, and also from the revision of previous results where land‐snail faunas were observed to be decreasing in diversity along the elevation gradient of this mountain (Tattersfield, Warui, Seddon, & Kiringe, 2001).

Even if different groups of life‐forms and phytogeographic affinities have similar but slightly modified richness‐elevation patterns with total species, the proportion of each component varies very much along the elevation gradient (Figure 2). Taller life‐forms like trees and shrubs are confined to the lower elevations, and similar patterns of lianas and ferns are coupled to that of trees (Carpenter, 2005; Kluge et al., 2017), leading to a significant decrease in the proportion of woody plants along the elevation gradient, and this reflects physiological adaptations to high elevation and alpine environments (Kluge et al., 2017; Körner, 2003). Analogous to most mountains of the world (Steinbauer et al., 2016), such as Andes (Kessler, 2000), Himalayas (Kluge et al., 2017; Vetaas & Grytnes, 2002), and Hengduan Mountains (Zhang, Zhang, Boufford, & Sun, 2009), endemic species are confined to high elevations in the tropical African mountains (Hedberg, 1969; Morton, 1972). The endemic species of Mount Kenya appear above 1,800 m, increase along the elevation gradient, and decrease in the high elevations with the highest richness at ca. 3,900 m a.s.l. (Figure 3). Above heath zone of Mount Kenya, the vegetation becomes dominated by giant rosette plants *Dendrosenecio* spp. and *Lobelia* spp., named Afro‐alpine vegetation with the elevation from ca. 3,500 to 4,800 m a.s.l., with numerous endemic species, such as *Carduus schimperi* subsp. *platyphyllus*, *Dendrosenecio keniensis,* and *Lobelia gregoriana* (Coe, 1967; Niemelä & Pellikka, 2004; Zhou et al., 2018).

An increase in the elevation range of occurrence of species in an assemblage with increasing elevation is explainable as a consequence of individuals having to be able to withstand a broader range of climatic conditions at higher elevations (Fernández & Vrba, 2005; Gaston & Chown, 1999; Morin & Lechowicz, 2011). Herbaceous species can always adapt to new climatic conditions 2 to 10 times faster than woody species for the latter have longer reproductive cycles and tend to accumulate genetic changes at slower rates (Smith & Beaulieu, 2009). Therefore, compared with woody species, herbaceous species have significantly higher elevation ranges (Figure 3a), which can be reflected in some exotic herbs with strong invasiveness (Giorgis et al., 2016; Molina‐Montenegro & Naya, 2012; Yang et al., 2018). Some studies have tried to divide species in an assemblage into different components, such as tropical and temperate species to investigate their differences in elevation range (Feng et al., 2016), while, few studies have divided species into different groups depending on their dispersal regions. Janzen (1967) proposed the influential hypothesis, stating that tropical mountains are physiologically higher than temperate mountains, namely, that elevational range sizes of organisms get smaller on mountains at decreasing latitudes (McCain, 2009). That is to say, the plant species restricted to the tropical regions (such as tropical East African species or endemic species in Mount Kenya) have smaller elevation ranges than the widely distributed species in the world (Figure 3b).

A strong support for the range‐elevation relationships predicted by elevation Rapoport's rules (ERR) was observed in total and herbaceous species (including climbers and herbs), as well as in different phytogeographic affinities (Figure 5a,d,h–l). However, the decreasing trend of the mean elevation ranges in high elevations has been detected in lycophytes and ferns and woody species (including trees, shrubs, and lianas) (Figure 5b,c,e–g). Bhattarai and Vetaas (2006) observed the similar decreasing trend of trees above 1,500 m a.s.l., with narrow elevational ranges at both ends of the gradient and a wider elevation range in the middle, and the explanation for this shift was boundary effects. Feng et al. (2016) came to a similar conclusion that boundary effects such as environmental or climatic conditions could cause a trend of decreasing of average range size at high elevation regions. Considering that total and herbaceous species showed support for the ERR with increasing trend of the range size‐elevation relationship, we speculate that the boundary effect did not notably impact the patterns of lycophytes and ferns and woody species. Actually, the proportion of narrowly distributed and endemic species increasing along the elevation gradient might impact the average elevation range size‐elevation relationship of species assemblages (Vetaas & Grytnes, 2002). In Mount Kenya, a high proportion of narrowly distributed species emerged in the high elevation gradient successively, such as *Phlegmariurus saururus* of lycophytes, *Dendrosenecio keniodendron*, *Erica trimera* subsp.* kenensis,* and *Helichrysum citrispinum*of woody species.

## 5 CONCLUSIONS

This study firstly tested the elevational Rapoport's rule by dividing all plants into different components, after comprehensively mastering the plant diversity of a tropical African mountain. The elevation range of the herbaceous species was significantly higher than the woody species, and the elevation range of the narrowly distributed species was significantly lower than the widely distributed species. These indicate that the widely distributed herbaceous species have broad elevation range size because they can probably withstand a broader range of climatic conditions, thus can possibly be more applicable to elevational Rapoport's rule. Therefore, we concluded that this rule is not consistent among different organisms (such as different life‐forms) in the same region.

## CONFLICT OF INTEREST

None Declared.

## AUTHOR CONTRIBUTIONS

Y.Z. and A.C.O. conceived and wrote the paper. Y.Z., G.H., and Q.W. provided the data. Y.Z., A.C.O., A.W.N., and B.H.B. analyzed the data. H.X., G.M., and Q.W. provided the idea. All authors reviewed the manuscript.

## Data Availability

Data are available via the Dryad Digital Repository: https://doi.org/10.5061/dryad.m6q87k1.
